# Supplementary material for: Global branches and local states of the human gut microbiome define associations with environmental and intrinsic factors
Source: Nat Commun. 2023 Jun 20;14:3310. doi: 10.1038/s41467-023-38558-7 (PMC10282066; doi:10.1038/s41467-023-38558-7)
Supplement: Supplementary file 1 — Supplementary Information [file 41467_2023_38558_MOESM1_ESM.pdf]

Supplementary Information

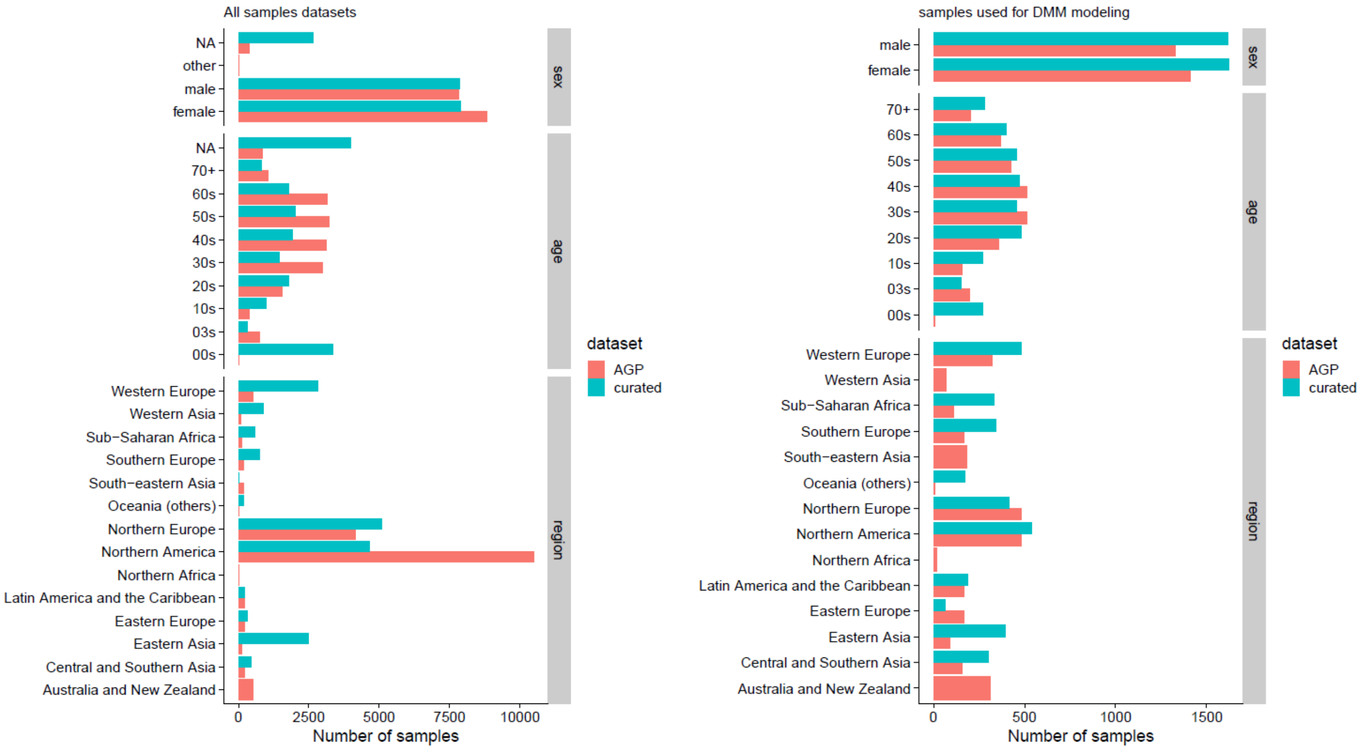

Figure S1 : Number of samples from the AGP and CuratedMetagenomicsData included (left panel) and used for DMM modelling (right panel) as a function of sex, age and geographic region. For the AGP dataset, geographic region corresponds to the region of birth. For age category, "00s" corresponds to 0 to 3 years old, "03s" corresponds to 3 to 10 years old.

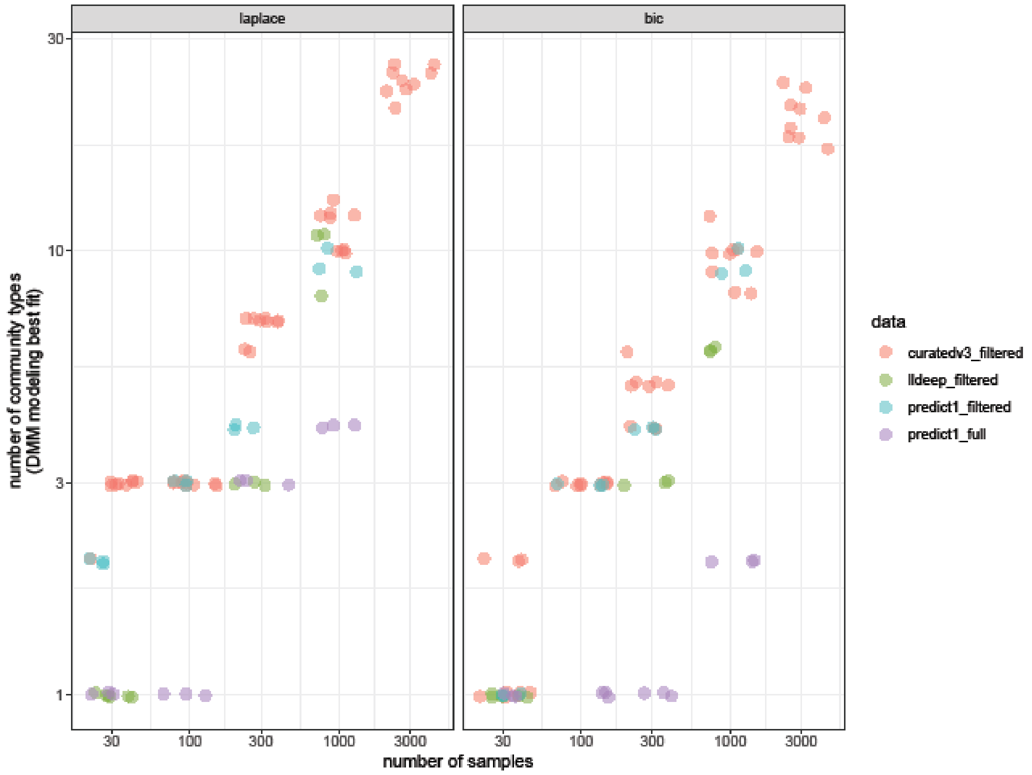

Figure S2: Number of partitions detected using the best fit for DMM modeling by Laplace or Bayesian information criterion (BIC) as a function of the number of samples, feature filtering and study cohorts. Random samples of varying size either from the full CMD or from the PREDICT1 and LifeLines-DEEP sub-cohorts were shown. Dots are jittered for better readability.

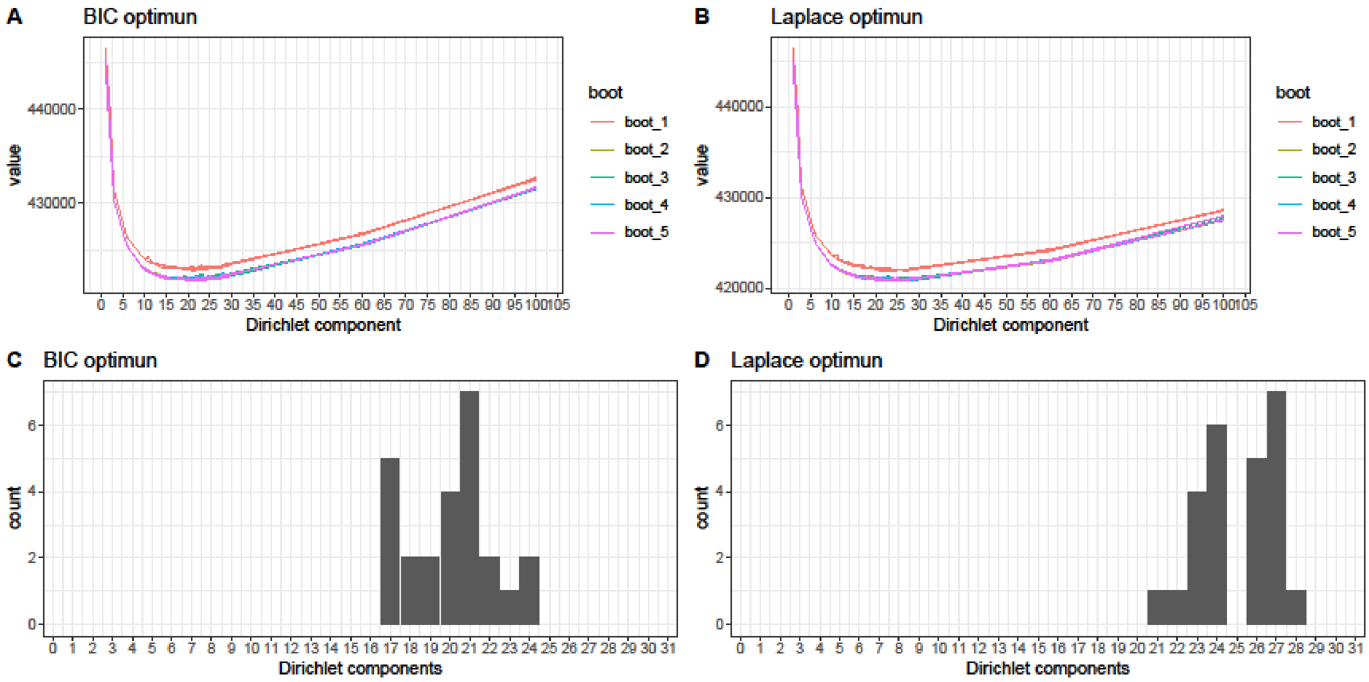

Figure S3: BIC (A) and Laplace (B) criteria as a function of the number of Dirichlet components throughout five subsampling in CuratedMetagenomicsData. Distribution of the optimal number of DMM components detected using (C) BIC and (D) Laplace throughout computational seeds and subsampling sets. A consensus value of 24 components was used for further analyses using majority vote.

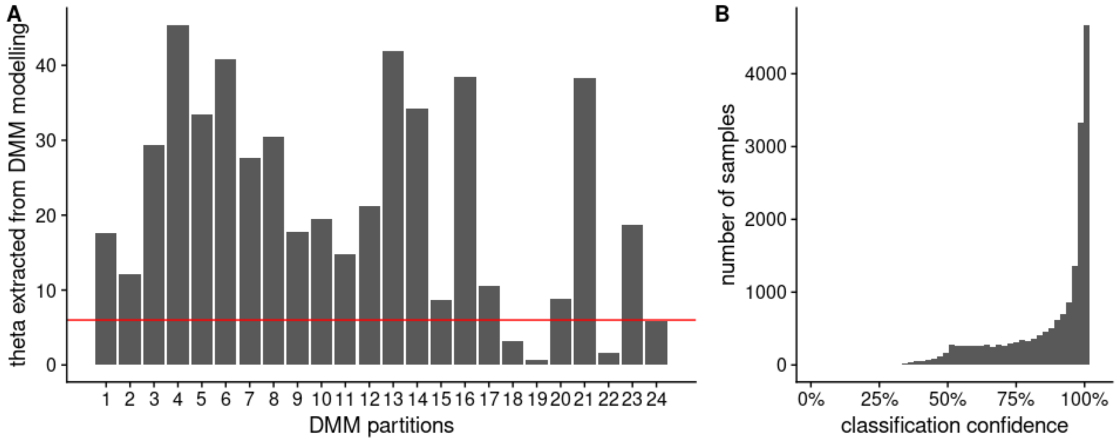

Figure S4: (A) DMM partitions homogeneity (theta) extracted from modelling and (B) classification confidence distribution. The red line depicts the theta obtained in case of no partitioning.

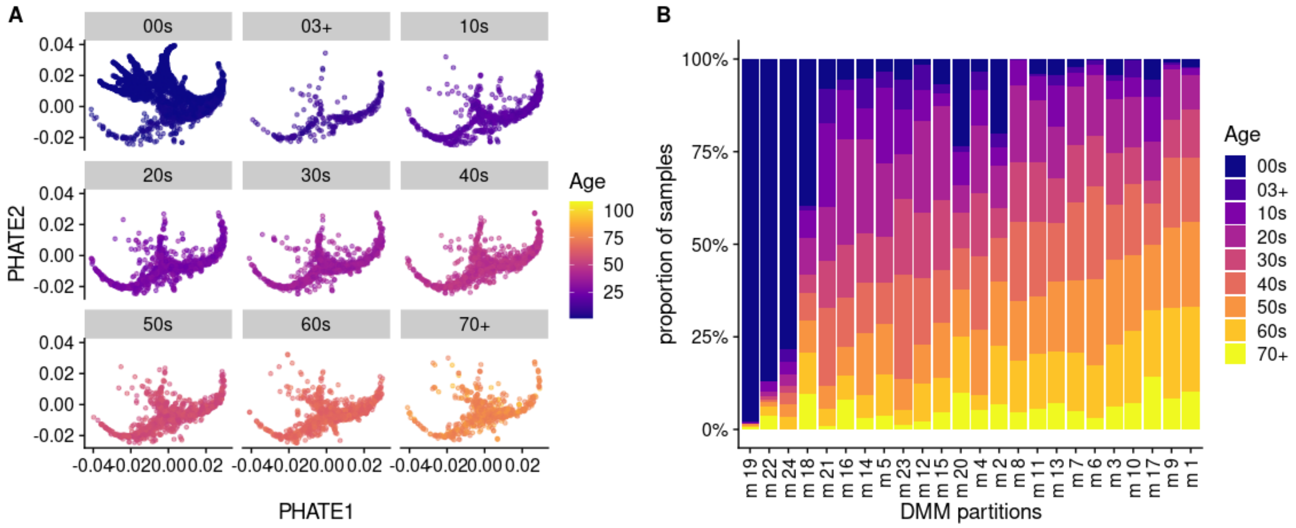

Figure S5: (A) PHATE based scatter plot faceted by age category and (B) sample proportion per age category and per DMM partitions

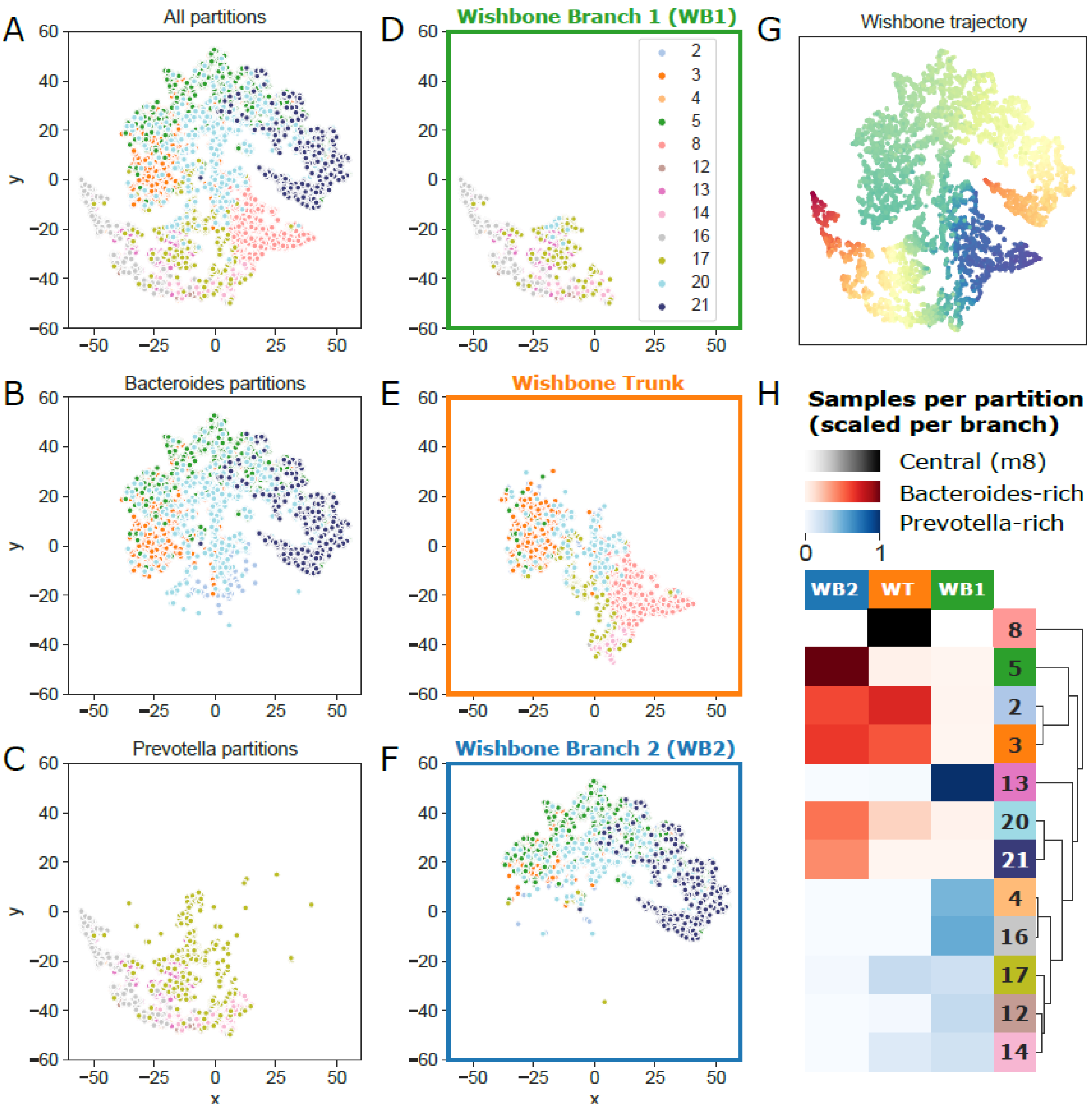

Figure S6 . Partitions along the trajectories obtained from a pseudo-time analysis using Wishbone. Partitions are labelled on a t-SNE dimensionality reduction representation for all samples (A), and showing sample subsets corresponding to (B) the *Bacteroides* partitions (m2, m3, m5, m20, m21) or (C) the *Prevotella* partitions (m4, m12, m13, m14, m16, m17), as well as for samples assigned by Wishbone to the trajectory trunk (i.e root) (E) or to trajectory ends towards the *Prevotella*-rich (D) or *Bacteroides*-rich (F) samples. The Wishbone trajectory consist of sample-associated values shown by a blue-to-red gradient (G). The number of samples belonging to the different partitions are colored (after scaling by subtracting the minimum and dividing by the maximum) for each Wishbone branch (WB1 and WB2) and trunk (T), in black for the central partition (m8) or in blue and red for the partitions enriched in the *Prevotella* and *Bacteroides* branches (H), respectively.

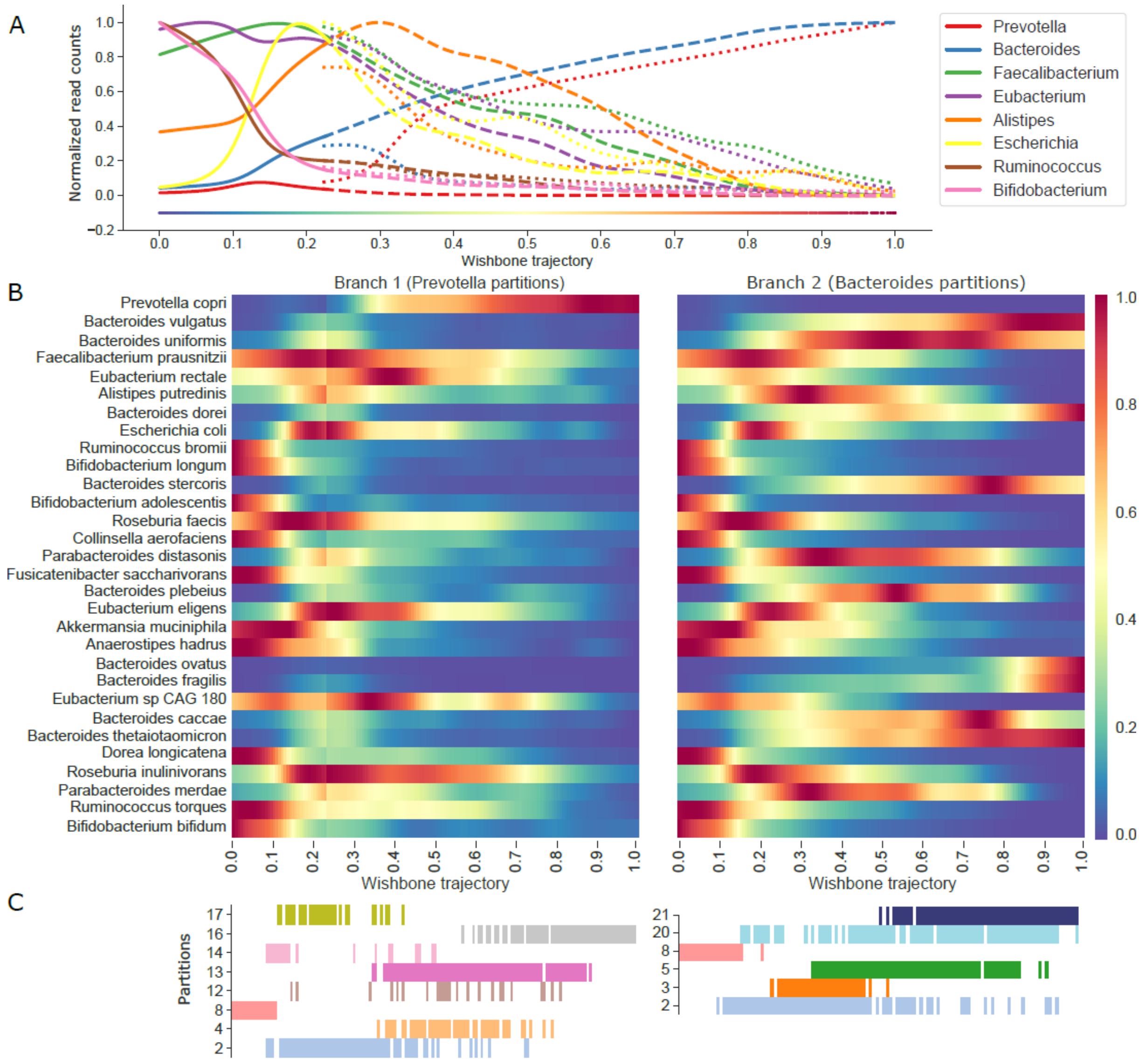

Figure S7. Normalized read abundances for the top 30 top species and their genera along the Wishbone trajectory. (A) Genera abundances along the trajectory starting from the m8 root (plain lines) and bifurcating either into the branch composed of samples enriched in *Prevotella* (short-dash lines) or into the branch composed of samples enriched in *Bacteroides* (long-dash lines). Underlying colors represents the position of the binned samples along this trajectory. (B) Heatmaps showing top-30 species read abundances, normalised for each of 150 equal-sized bins of samples along the Wishbone trajectory. The trajectory detected from a unique trunk and to the end of two detected branches thus visits the samples enriched in *Prevotella* (left panel) and in *Bacteroides* (right panel). (C) Partitions of the samples in each of the 150 equal-sized bins along the Wishbone trajectory, separately for the samples enriched in *Prevotella* (left panel) and in *Bacteroides* (right panel). For each bin, the visited partitions are indicated by vertical bars (colored as in Fig S6). If more than 90% of the samples of a bin belong to the same partition, a single bar is shown. Otherwise, all partitions represented by at least 10% of the bin samples are shown

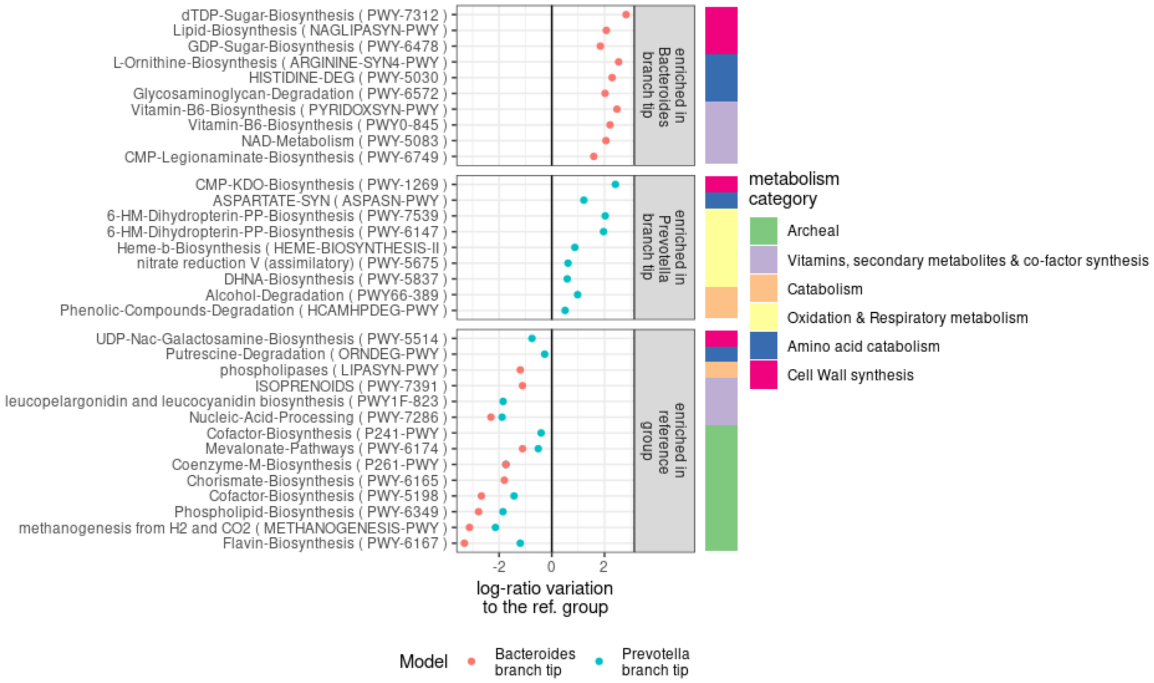

Figure S8: Microbial functions differentially associated with gut microbiome branch tips. The dot plot show the 10 pathways found most enriched by differential abundance modeling for the partitions representing the tips of the Prevotella (m16) and Bacteroides (m21) branches. Both models use partition M8 as a reference group: the 10 pathways found most enriched in the samples of this reference partition are also shown. Dot color account for branch tip dominated respectively by Bacteroides and Prevotella. For each pathway across samples of each branch tip, relative abundances are expressed in terms of log ratios (using 21 "nucleotide biosynthesis" pathways as a denominator) with the arithmetic mean shown. Each pathway was classified within a metabolism category extracted from MetaCyc database.

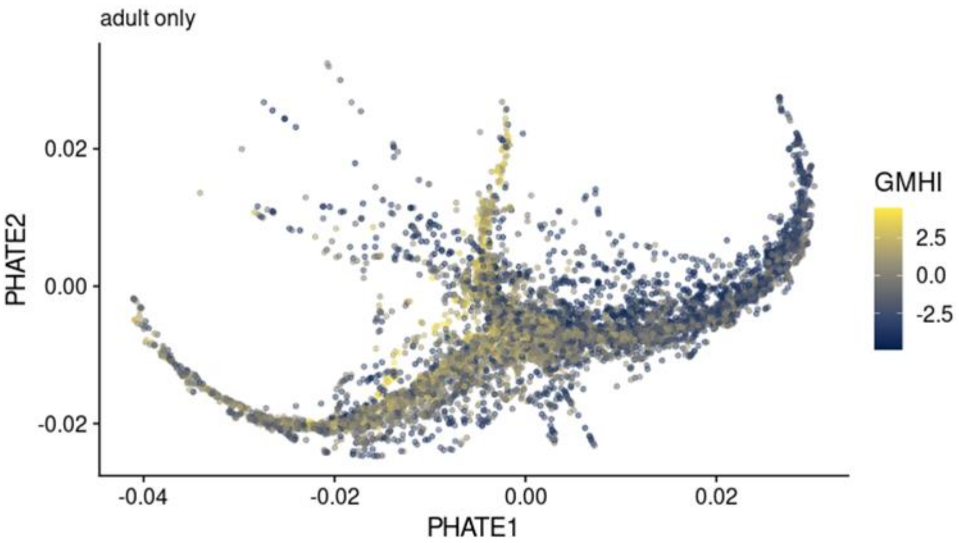

Figure S9. Gut Microbiota Healthy Index (GMHI) in adults on PHATE scatter plot based on Curated Metagenomics Data.

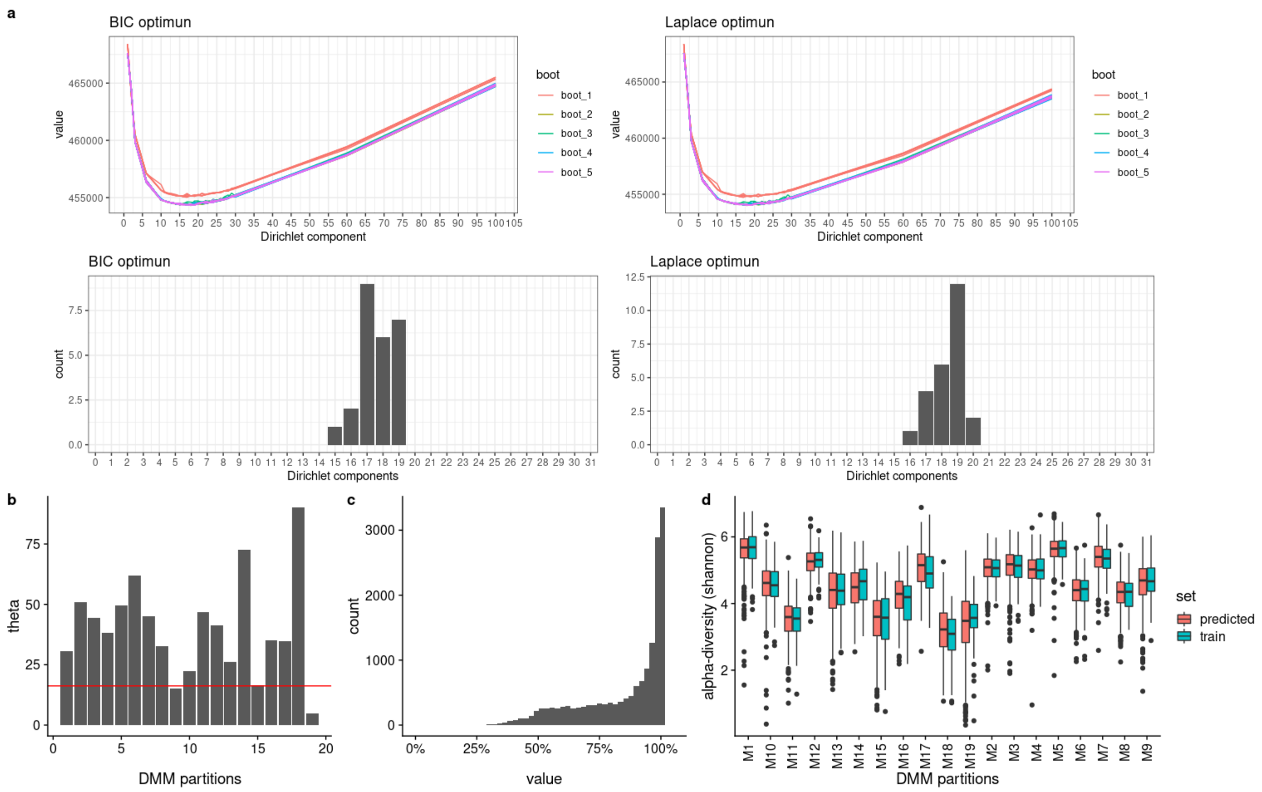

Figure S10: (A) BIC and Laplace criteria as a function of the number of Dirichlet components throughout five subsampling in the American Gut project dataset. Distribution of the optimal number of DMM components using BIC and Laplace criteria throughout computational seeds and subsampling sets. (B) DMM partitions homogeneity (theta) extracted from modelling and (C) classification confidence distribution for the American Gut Project dataset. D) Boxplot for alpha diversity (Shannon's) as function of DMM partitions per train set and predicted set (n=16,021 samples). The box bounds the IQR divided by the median, and whiskers extend to a maximum of 1.5 × IQR beyond the box. Dots are sample data points A consensus value of 19 components was used for further analyses using majority vote.

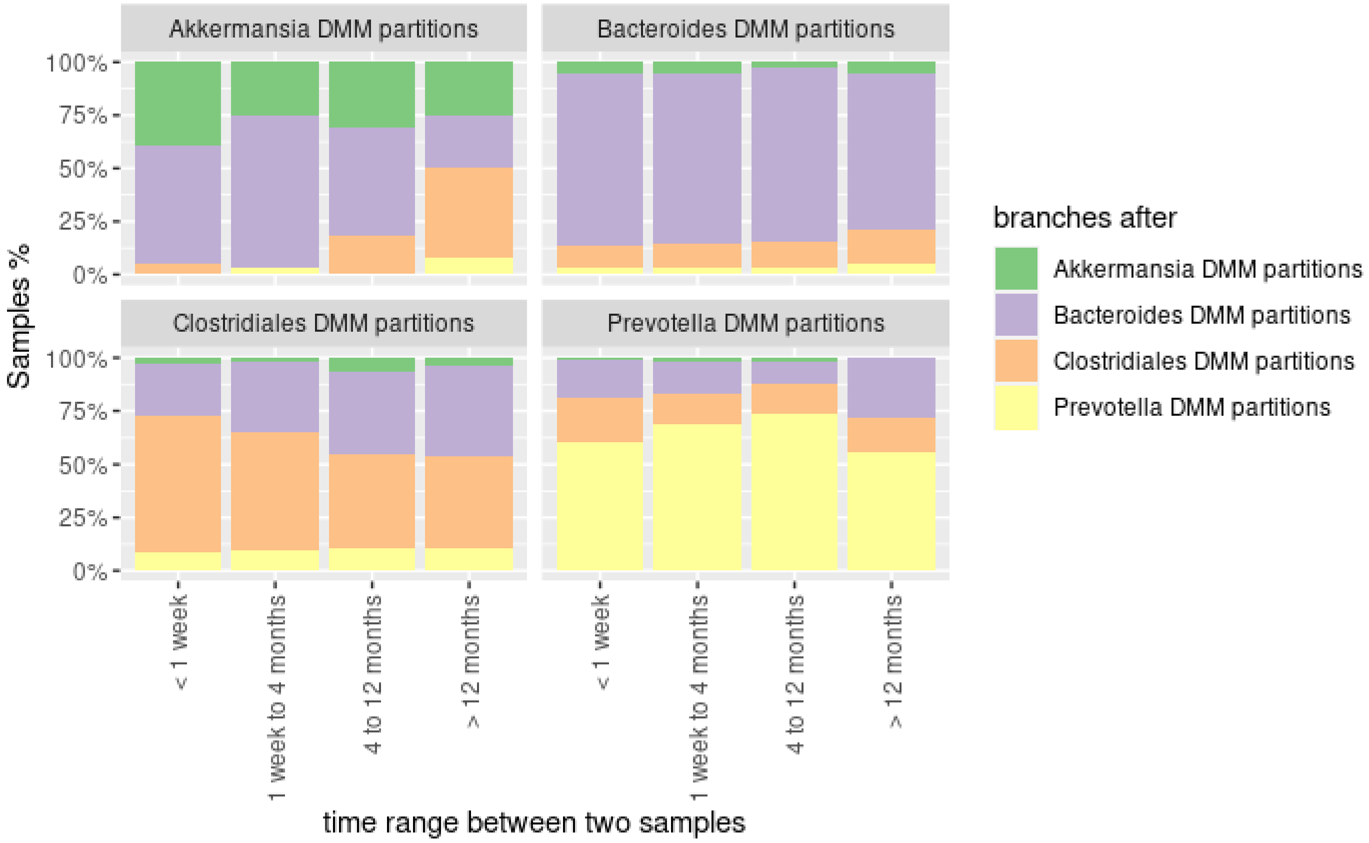

Figure S11: Subject proportion assigned to DMM partitions before and after as a function of time range between two samples. Each panel title corresponded to a DMM partitions before, and color account for DMM partitions after a range time (x-axis). For instance, 25% of individuals assigned to Clostridiales DMM partitions were assigned to a Bacteroides DMM partition within a week.

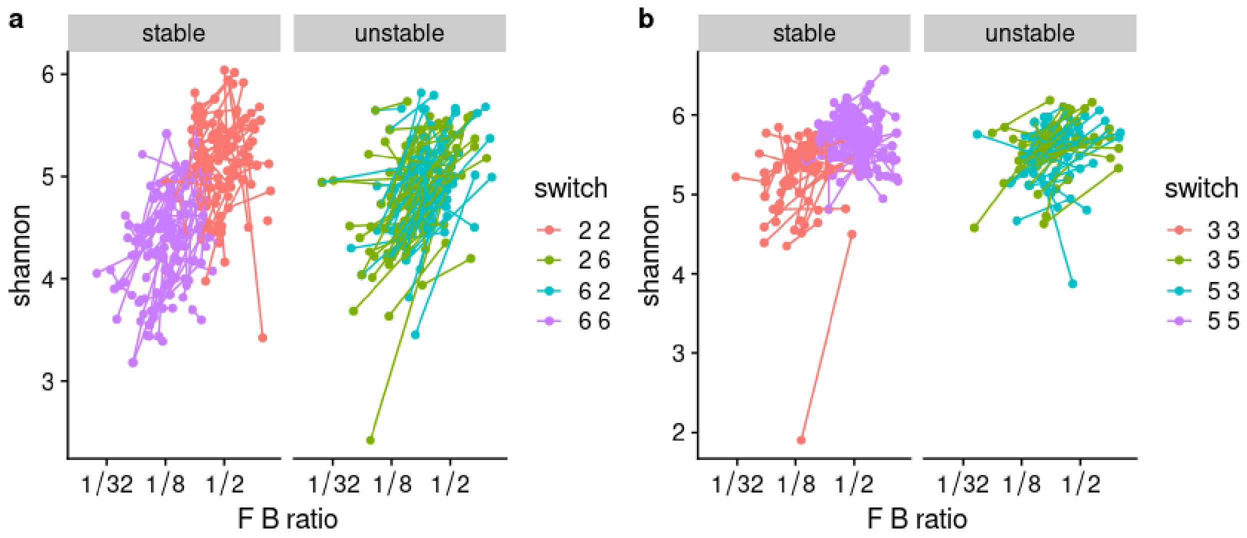

Figure S12: alpha-diversity variation as a function of *Faecalibacterium*:*Bacteroides* variation among (a) M2/M6 and (b) M3/M5 stability assessment. "2 2" indicated an individual that remains in partition M2 while "2 6" indicated an individual that switched from partition M2 to M6
