# Supplementary material for: Global branches and local states of the human gut microbiome define associations with environmental and intrinsic factors
Source: Nat Commun. 2023 Jun 20;14:3310. doi: 10.1038/s41467-023-38558-7 (PMC10282066; doi:10.1038/s41467-023-38558-7)
Supplement: Supplementary file 3 — Description of Additional Supplementary Files [file 41467_2023_38558_MOESM3_ESM.docx]

**Description of Additional Supplementary Files Document**

Supplementary Dataset 1 - Permanova test between training and remaining set for each partition in CMD dataset. Tests were two-sided and FDR adjusted.

Supplementary Dataset 2: Spearman's rank correlation test between genus and Shannon index. Tests were two-sided and FDR adjusted.

Supplementary Dataset 3. Differential analysis of bacterial species between root and tips and branches using Mann-Whitney on CLR abundance. Tests were two-sided and FDR adjusted.

Supplementary Dataset 4. Odd ratio estimates using multinomial regression per predictors and per partitions in AGP database
